# Topical Application of Human Umbilical Cord-Derived Mesenchymal Stem Cell Exosomes for a Deep Second-Degree Scald Burn Wound: A Case Report

**DOI:** 10.7759/cureus.107198

**Published:** 2026-04-16

**Authors:** Connie Zhi-Chen Hung, Tsai-Hua Chung, Wen-Hsien Lin

**Affiliations:** 1 Dermatology, Morning Glory Lab Clinic, Taipei, TWN; 2 Medical Education, BIONET Therapeutics Corp., Taipei, TWN; 3 Bioinformatics, Genetics Generation Advancement (GGA) Corp., Taipei, TWN

**Keywords:** case report, cell-free therapy, exosomes, mesenchymal stem cells, scald burn, wound healing

## Abstract

Conventional treatments for deep second-degree scald burns are frequently associated with prolonged healing times and hypertrophic scarring. Exosomes derived from mesenchymal stem cells represent a promising cell-free therapeutic strategy owing to their capacity to promote tissue regeneration. This report describes a 46-year-old woman with an unremarkable medical history who sustained a deep burn to her left thigh. The injury, classified as deep second-degree per American Burn Association (ABA) standards and involving 1% of the total body surface area (TBSA), significantly hindered her daily activities. Although not life-threatening, the localized pain and wound extent caused substantial functional impairment. Following one week of standard wound care without significant clinical improvement, a topical formulation containing human umbilical cord-derived mesenchymal stem cell exosomes (UC-MSC-Exos; ExoVia, BIONET Therapeutics Corp., Taipei, Taiwan) was applied once daily for nine consecutive days. Progressive granulation tissue formation and accelerated re-epithelialization were observed throughout the treatment period. Complete wound closure was achieved by day 18 post-injury, and the one-month follow-up demonstrated excellent skin quality with minimal scarring. No adverse reactions occurred during the treatment course. These preliminary findings suggest that topical application of UC-MSC-Exos could potentially serve as a well-tolerated adjunctive therapy associated with favorable healing for deep second-degree scald burns. Further investigation through larger, controlled clinical trials is warranted to validate these preliminary observations.

## Introduction

Burn injuries represent a significant global health burden. According to the World Health Organization (WHO), an estimated 11 million people seek medical attention annually worldwide. Among these, deep second-degree scald burns, characterized by damage extending through the epidermis into the deeper dermis, pose particular clinical challenges. The healing process for such injuries is a complex biological sequence traditionally divided into four overlapping phases: hemostasis, inflammation, proliferation, and remodeling. This healing process is inherently prolonged and susceptible to complications, including infection and dysregulated inflammation, which frequently result in hypertrophic scarring and functional impairment [1]. Current standard treatments, such as topical silver sulfadiazine and various wound dressings, are associated with several known disadvantages. Silver sulfadiazine, for instance, has been associated with delayed re-epithelialization, while extensive wounds may necessitate autologous skin grafting, thereby creating secondary donor site morbidity [2]. Consequently, there exists a substantial unmet need for novel therapeutic strategies capable of accelerating wound closure while optimizing the quality of tissue regeneration.

Despite being inherently safer than many other stem cell types, mesenchymal stem cells (MSCs) still carry inherent risks such as immunogenicity and tumorigenicity and logistical challenges related to storage and administration [3,4]. Accumulating evidence indicates that the therapeutic benefits of MSCs are predominantly mediated through paracrine mechanisms, with extracellular vesicles, particularly exosomes, as the principal functional mediators [5,6]. As endogenous nanocarriers of bioactive molecules, including proteins, lipids, and nucleic acids, exosomes play a pivotal role in intercellular communication and modulate critical processes in wound healing, such as inflammation, angiogenesis, and tissue remodeling [7].

Exosomes derived from human umbilical cord MSCs (UC-MSCs) are particularly attractive for therapeutic applications due to their high yield, low immunogenicity, and potent regenerative capacity [8,9]. Although preclinical studies have demonstrated the efficacy of UC-MSC exosomes in promoting wound healing across various models, clinical evidence supporting their application in human deep second-degree scald burns remains limited [10]. This case report documents the topical application of a standardized, quality-controlled UC-MSC exosome formulation as an adjunctive therapy for a deep second-degree scald burn, with the objective of providing preliminary clinical insights into its safety and potential efficacy.

## Case presentation

A 46-year-old woman with an unremarkable medical history presented for evaluation. She had no history of diabetes, hypertension, autoimmune disorders, or other conditions known to affect wound healing. Additionally, she reported no known drug allergies and denied tobacco or alcohol use.

On June 6, 2025, the patient sustained a thermal injury to her anterior left thigh following accidental contact with boiling soup. She immediately initiated first aid by applying cool running water to the affected area for approximately 15 minutes prior to seeking medical attention.

**Figure 1:**
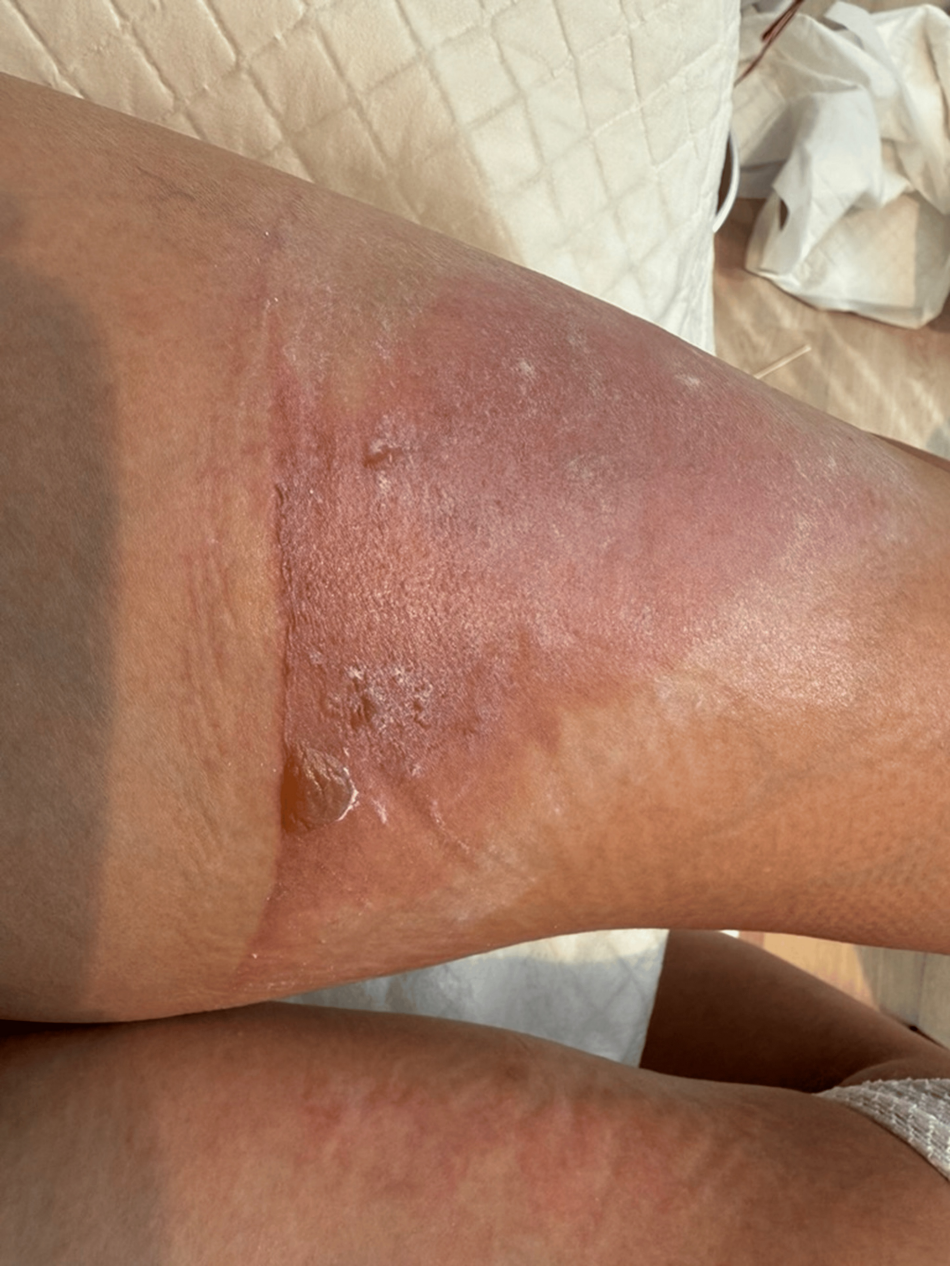
Clinical presentation on the day of injury (day 0). The deep second-degree scald burn on the anterior left thigh demonstrates characteristic erythema and a mottled wound bed with areas of epidermal loss

Physical examination revealed a deep burn measuring approximately 15×8 cm. Based on the rule of palms, this area corresponded to approximately 1% of the total body surface area (TBSA). Clinical depth assessment was performed in accordance with the American Burn Association (ABA) criteria; the wound bed exhibited a characteristic moist, mottled red-and-white appearance with ruptured bullae. Furthermore, diminished pain sensation to blunt pressure was noted, confirming a deep second-degree injury (Figure 1).

Therapeutic intervention

The exosome formulation employed in this case was ExoVia (BIONET Therapeutics Corp., Taipei, Taiwan), containing human UC-MSC-derived exosomes. This product was manufactured in a facility compliant with Good Manufacturing Practice (GMP) standards. The raw material is registered with the Taiwan Food and Drug Administration (TFDA) and has been assigned an International Nomenclature Cosmetic Ingredient (INCI) name, ensuring standardized identification and regulatory compliance. Characterization was performed in alignment with the minimal information for studies of extracellular vesicles (MISEV) guidelines suggested by the International Society for Extracellular Vesicles (ISEV): transmission electron microscopy demonstrated spherical morphology reflecting the native state of the vesicles, with diameters ranging from 50 to 120 nm; nanoparticle tracking analysis indicated a concentration of 3.33×10^9^ particles/mL; and Western blot analysis confirmed the expression of canonical exosome markers CD9, CD63, CD81, and TSG101. The exosome product was supplied in 3-mL vials containing 1×10^10^ exosome particles, maintained under frozen storage for up to one year. In accordance with the manufacturer's specifications, the vial was thawed at room temperature, reaching a liquid form immediately prior to each topical application.

During the first week post-injury (days 0 through 6), the patient received standard wound care consisting of initial cleansing and debridement, followed by daily irrigation with normal saline. The wound was treated with topical antimicrobial silver sulfadiazine cream and covered with non-adherent sterile gauze dressings, with dressing changes performed once daily. No systemic antibiotics were administered, and pain management was limited to as-needed analgesics. Throughout this period, the wound exhibited characteristic features of the acute inflammatory phase, including pain and substantial exudate production.

After six days of standard clinical treatment, the wound showed no significant improvement. Consequently, adjunctive therapy with ExoVia was initiated on day 7. One entire vial of the exosome formulation, containing 1×10^10^ exosome particles, was evenly distributed over the wound bed once daily following wound cleansing and prior to the application of the standard ointment and dressing. This regimen was continued for nine consecutive days, concluding on day 15.

Treatment course and outcomes

Wound progression was systematically documented through serial photography at predetermined intervals.

**Figure 2:**
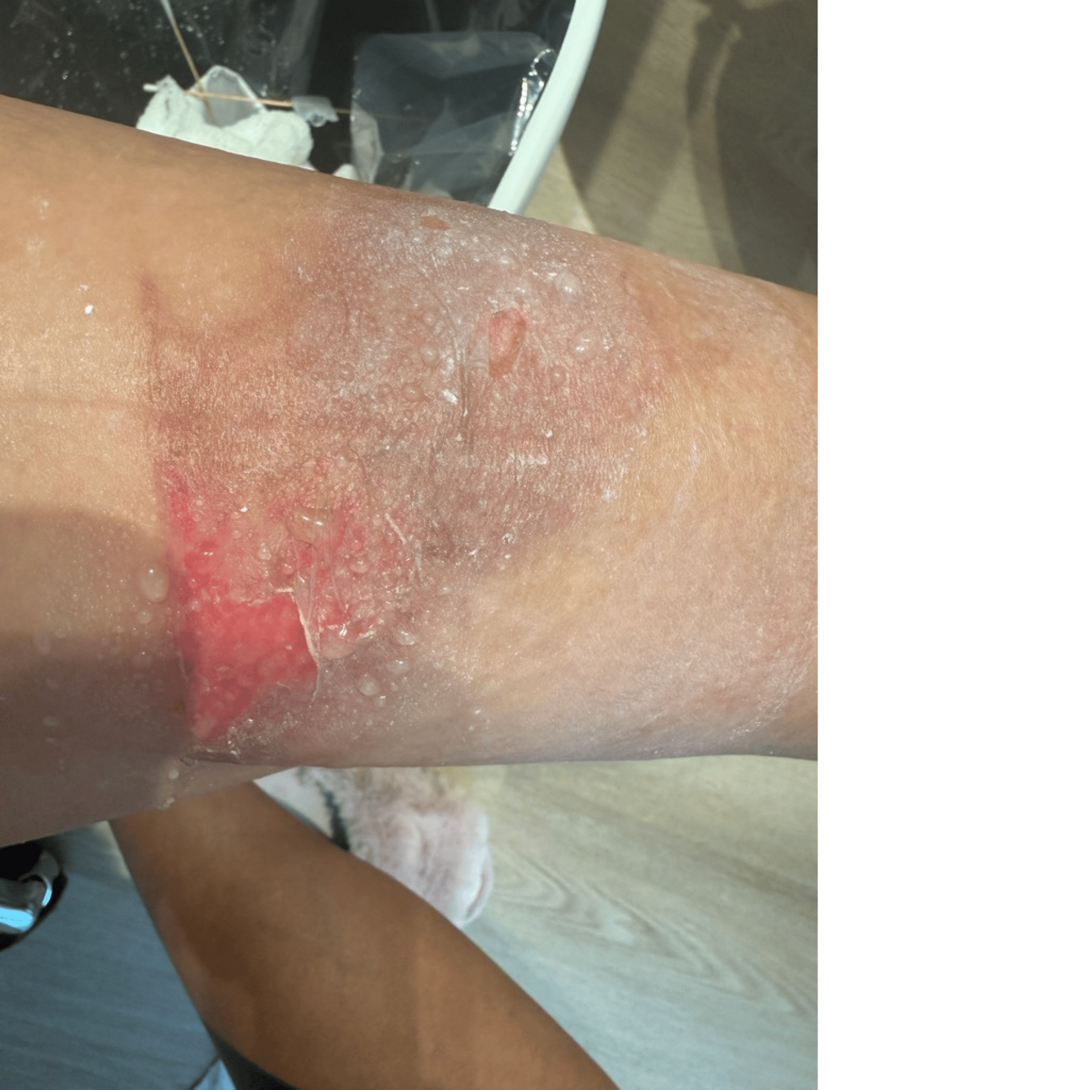
Wound appearance on day 7, the first day of ExoVia application. Early granulation tissue is visible at the wound periphery, with reduction in surrounding erythema

On day 7, coinciding with the initiation of exosome therapy, light red granulation tissue became evident at the wound margins, signifying the onset of the proliferative phase of wound healing (Figure 2).

**Figure 3:**
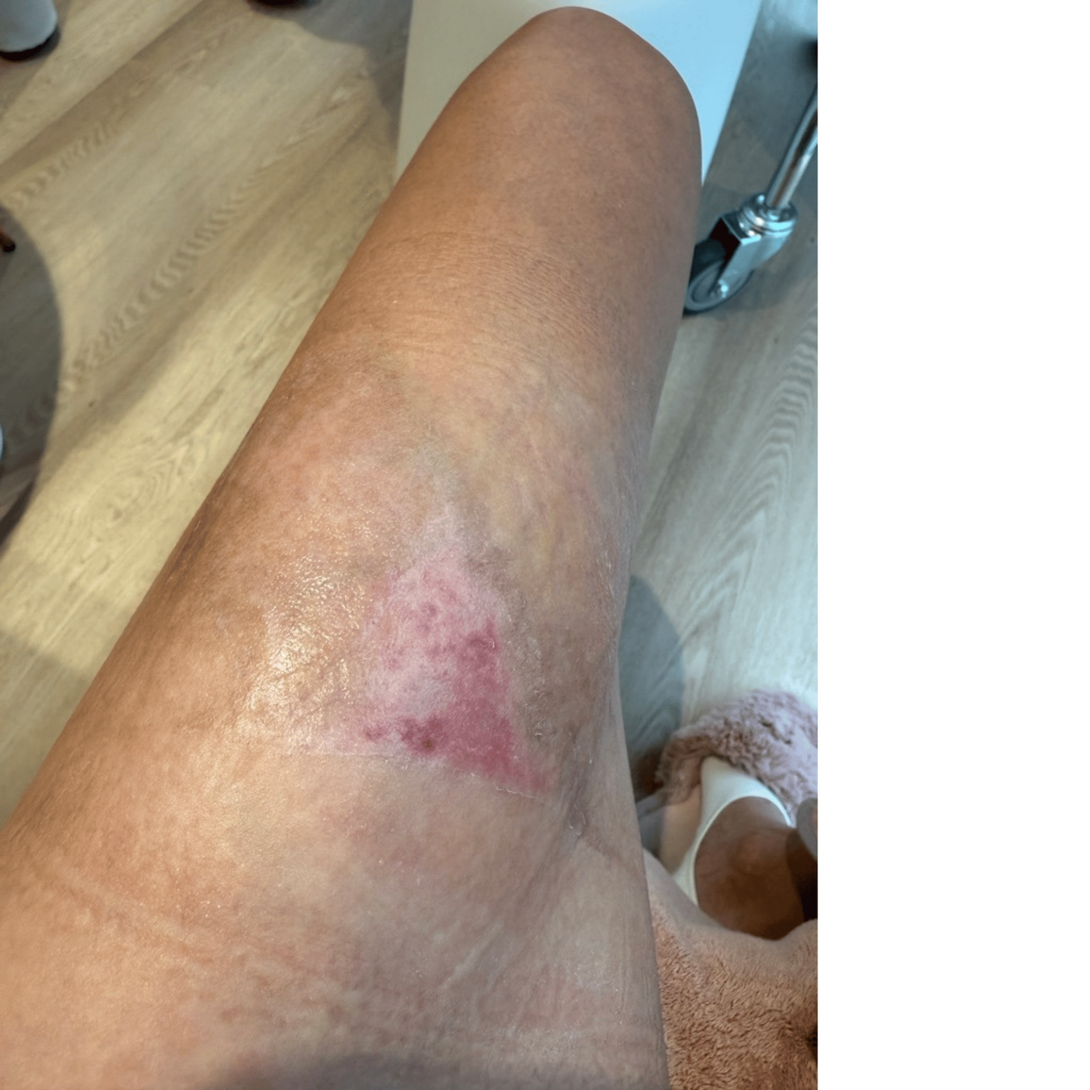
Wound appearance on day 10. Significant wound contraction is evident, with healthy granulation tissue covering the majority of the wound bed and progressive re-epithelialization at the margins

By day 10, healthy granulation tissue had extended to cover the majority of the wound bed, and substantial wound contraction was apparent. Active re-epithelialization was observed, with new epithelial tissue advancing from the wound margins (Figure 3).

**Figure 4:**
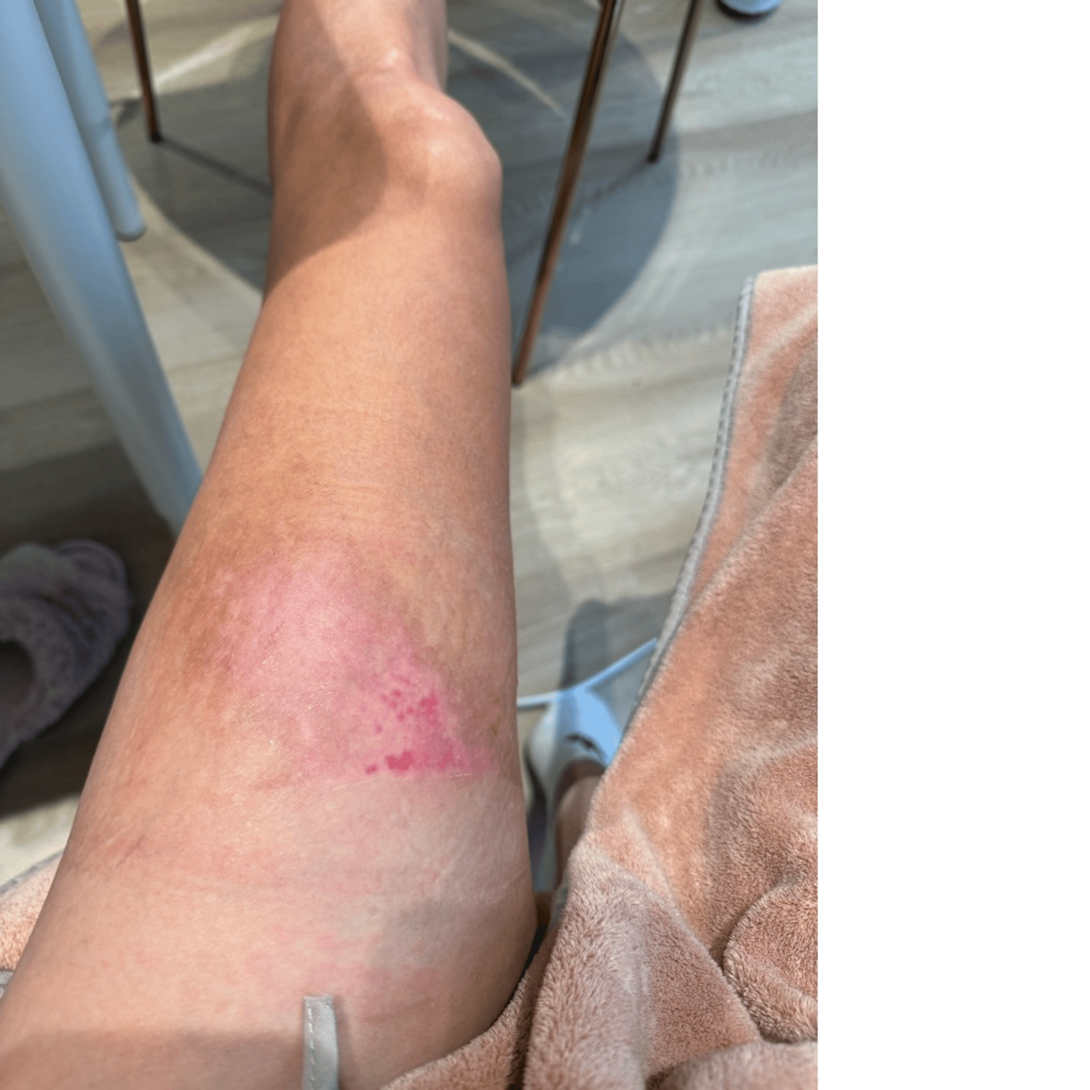
Wound appearance on day 15, upon the completion of the nine-day ExoVia treatment course. The wound is nearly healed, with minimal residual erythema

Upon the completion of the exosome treatment on day 15, the wound demonstrated near-complete closure with only minimal residual erythema centrally (Figure 4).

**Figure 5:**
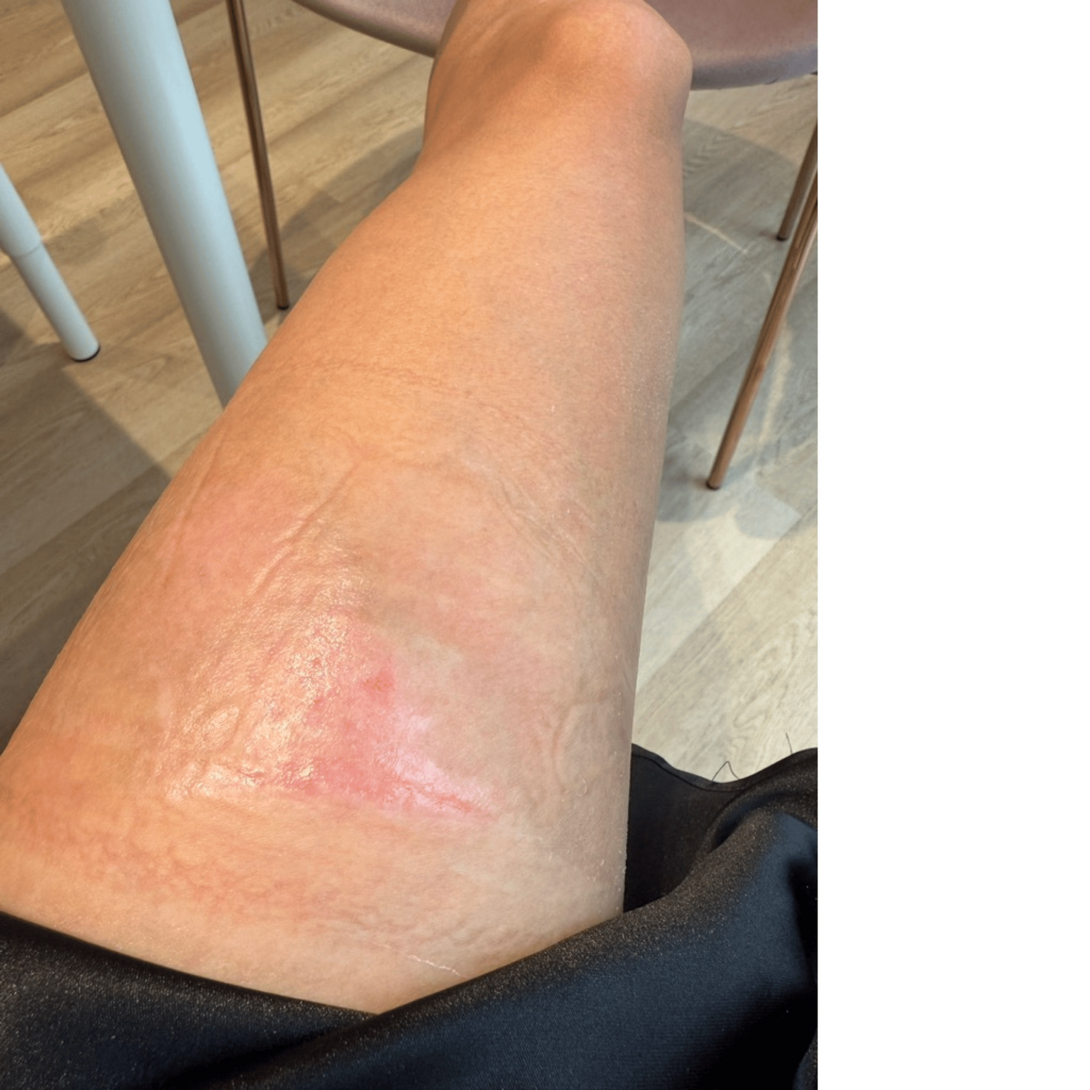
Wound appearance on day 18. Complete wound closure is achieved, with new epithelial tissue covering the entire wound area

Assessment on day 18 confirmed complete wound closure, with new epithelial tissue providing full coverage of the previously injured area (Figure 5). Throughout the treatment course, the patient experienced no local adverse effects, including pruritus or contact dermatitis, nor any systemic reactions.

**Figure 6:**
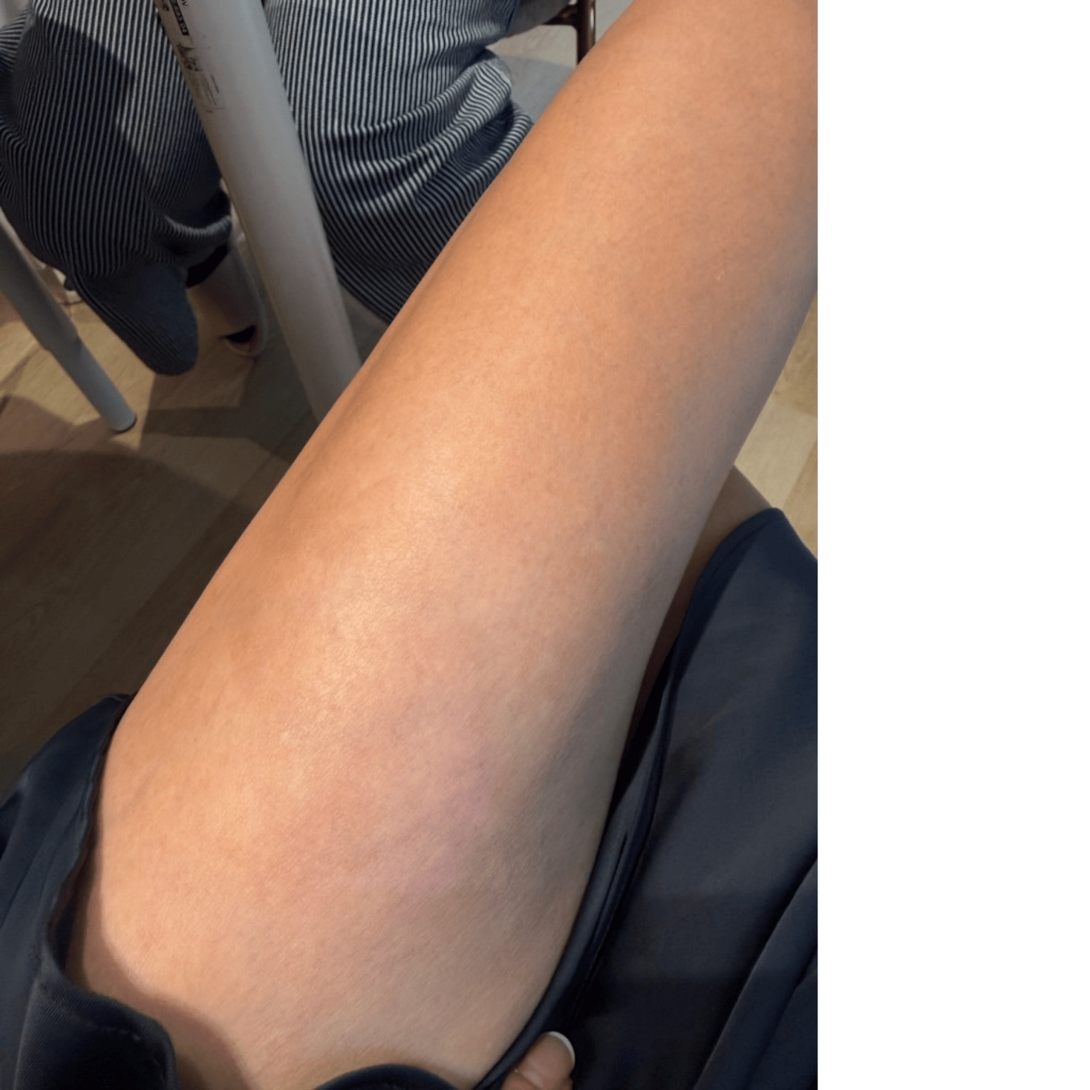
One-month follow-up (day 35). Excellent cosmetic outcome is observed, with skin color approaching that of surrounding tissue, good elasticity, and absence of hypertrophic scarring

At the one-month follow-up (day 35), the healed tissue demonstrated excellent cosmetic and functional outcomes. The skin exhibited good elasticity, minimal hyperpigmentation, and no clinical evidence of hypertrophic scarring (Figure 6).

The treatment timeline and corresponding wound status are summarized in Table 1.

**Table 1:** Treatment timeline and wound healing progression

Day post-Injury	Intervention	Clinical observations
Day 0	Immediate cooling; initiation of standard wound care	Deep second-degree burn with erythema and mottled wound bed (Figure 1)
Days 0-6	Standard wound care (debridement, burn ointment, sterile dressing)	Acute inflammatory phase characterized by pain and exudate
Day 7	Initiation of daily ExoVia application	Emergence of light red granulation tissue at wound margins (Figure 2)
Days 8-14	Continuation of ExoVia treatment	Substantial wound contraction; healthy granulation tissue covering the majority of the wound bed (Figure 3)
Day 15	Completion of ExoVia treatment (nine days total)	Near-complete wound closure with advanced re-epithelialization (Figure 4)
Day 18	Follow-up assessment	Complete wound closure with full epithelial coverage (Figure 5)
Day 35	One-month follow-up	Excellent cosmetic outcome; skin color approaching normal with no hypertrophic scarring (Figure 6)

## Discussion

This case report provides preliminary clinical evidence suggesting that the topical application of UC-MSC-derived exosomes, when used as an adjunct to standard wound care, may facilitate the healing of deep second-degree scald burns. Complete wound closure was achieved by day 18 post-injury. While this timeframe falls within the two-to-three-week range typically cited for deep second-degree burns treated with conventional methods, it is noteworthy that the wound bed exhibited healthy granulation tissue formation and the healed skin demonstrated minimal scarring at the one-month follow-up [11].

The mechanisms underlying the regenerative effects of UC-MSC exosomes are multifactorial, encompassing the coordinated modulation of inflammation, angiogenesis, and tissue remodeling [12]. During the initial inflammatory phase of wound healing, maintenance of an appropriate inflammatory balance is essential. Bioactive molecules contained within exosomes, including specific microRNAs such as miR-146a, have been shown to attenuate excessive inflammation by downregulating pro-inflammatory signaling cascades, thereby establishing a microenvironment conducive to tissue repair [13,14]. By the fourth day of exosome therapy, the clinical observation of reduced wound exudate and diminished erythema was consistent with such anti-inflammatory and pro-reparative effects of umbilical cord-derived mesenchymal stem cell exosomes (UC-MSC-Exos).

Additionally, exosomes are enriched with pro-angiogenic factors and regulatory miRNAs that promote neovascularization and enhance the migration and proliferation of epithelial cells [15,16]. The robust granulation tissue formation and accelerated re-epithelialization observed between days 10 and 15 in this case are concordant with these established functions, providing clinical support for the regenerative potential of exosome therapy.

Furthermore, MSC-derived exosomes may contribute to the mitigation of scar formation through the modulation of fibroblast activity and extracellular matrix deposition. Through the delivery of regulatory molecules such as miR-29a, exosomes can promote balanced collagen synthesis and degradation, thereby preventing the excessive fibrosis characteristic of hypertrophic scarring [17]. The favorable cosmetic outcome observed in this patient may be partially attributable to this regulatory mechanism.

However, it is imperative to acknowledge the significant limitations inherent in this study. As a single-case report without a control group, it is not possible to definitively attribute the clinical outcomes solely to the exosome therapy, nor can the contribution of standard wound care be fully isolated. Accordingly, these findings should be interpreted as preliminary and hypothesis-generating rather than as evidence of a causal relationship.

Furthermore, while previous human studies have reported the potential of MSC-derived exosomes in enhancing skin flap survival and chronic wound healing, standardized dosing and administration protocols for burn care remain to be established. Additionally, molecular analysis of wound exudate was not performed, which would be necessary to elucidate the underlying biological mechanisms directly. Moreover, we recognize that our one-month follow-up is insufficient for a comprehensive assessment of long-term outcomes; since hypertrophic scars often manifest between three and six months post-injury, extended longitudinal evaluation is warranted to monitor changes in pigmentation and scar maturation [18]. Finally, rigorous comparative studies against gold-standard treatments, such as silver sulfadiazine or modern bioactive dressings, are essential to validate the therapeutic superiority and cost-effectiveness of this regenerative approach.

Compared to cell-based therapeutic approaches, exosomes offer several practical advantages, including reduced immunogenicity, absence of tumorigenic risk, and greater amenability to standardization, storage, and topical application, rendering them an attractive platform for clinical translation [6,19]. The excellent safety profile observed in this case, utilizing a standardized and quality-controlled product, underscores the importance of product quality and appropriate administration route, particularly given that unapproved exosome formulations have been associated with adverse events in other contexts [15,20].

## Conclusions

This case report provides preliminary clinical evidence for the topical application of human UC-MSC-Exos as a potential adjunctive therapy in the management of deep second-degree scald burns. While the treatment was associated with complete wound healing and a favorable cosmetic outcome, we acknowledge that, as a single-case observation, these results are descriptive and cannot definitively establish superiority over conventional care.

Acknowledging the inherent limitations of a single-case observation, these results provide a compelling rationale for larger, prospective, randomized controlled trials. Such studies are essential to definitively establish the efficacy, safety, and optimal treatment protocols for exosome-based therapies in burn care. As the regulatory framework governing regenerative medicine products continues to evolve, rigorous clinical evidence will be paramount for the successful translation of this technology into routine clinical practice.
